# Effects of initial leaching for estimates of mass loss and microbial decomposition—Call for an increased nuance

**DOI:** 10.1002/ece3.9118

**Published:** 2022-07-31

**Authors:** Lovisa Lind, Andrew Harbicht, Eva Bergman, Johannes Edwartz, Rolf Lutz Eckstein

**Affiliations:** ^1^ Department of Environmental and Life Sciences – Biology Karlstad University Karlstad Sweden; ^2^ Fisheries and Ecosystem Sciences, Fisheries and Oceans Canada, Gulf Fisheries Centre Moncton New Brunswick Canada; ^3^ Population Ecology Division Fisheries and Oceans Canada, Bedford Institute of Oceanography Dartmouth Canada

**Keywords:** decomposition, leaching, microbial, Tea Bag Index

## Abstract

Decomposition is essential to carbon, nutrient, and energy cycling among and within ecosystems. Several methods have been proposed for studying litter decomposition by using a standardized and commercially available substrate. One of these methods is the Tea Bag Index (TBI) which uses tea bags (green and rooibos tea) incubated for ~90 days. The TBI is now applied all over the globe, but despite its usefulness and wide application, the TBI (as well as other methods) does not explicitly account for the differences in potential loss of litter mass due to initial leaching in habitats with large differences in moisture. We, therefore, studied the short‐term mass losses (3–4 h) due to initial leaching under field and laboratory conditions for green and rooibos tea using the TBI and contextualized our findings using existing long‐term mass loss (90 days) in the field for both aquatic and terrestrial environments. For both tea litter types, we found a fast initial leaching rate, which could be mistaken for decomposition through microbial activity. This initial leaching was higher than the hydrolyzable fraction given in the description of the TBI. We also found that leaching increased with increasing temperature and that leaching in terrestrial environments with high soil moisture (>90%) is almost as large as in aquatic environments. When comparing our findings to long‐term studies, we found that up to 30–50% of the mass loss of green tea reported as decomposition could be lost through leaching alone in high moisture environments (>90% soil moisture and submerged). Not accounting for such differences in initial leaching across habitats may lead to a systematic overestimation of the microbial decomposition in wet habitats. Future studies of microbial decomposition should adjust their methods depending on the habitat, and clearly specify the type of decomposition that the study focuses on.

## Graphical abstract

We found that both rooibos and green tea experienced a fast initial leaching rate, which eventually reached an asymptotic maximum. When comparing findings from our short‐term study to our to long‐term studies we found that up to 30‐50% of the mass loss reported as decomposition of green tea, could be lost through leaching alone in high moisture environments. Hence, not accounting for initial leaching will lead to a systematic overestimation of microbial decomposition and thus produce erroneous results when directly using mass loss values.

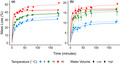

## 1 INTRODUCTION

The global carbon cycle describes the fluxes between carbon pools of different spheres on earth (Schlesinger & Andrews, 2000). While the total CO_2_ emission from soils represents one of the largest fluxes in the global carbon cycle (Schlesinger & Andrews, 2000), inland waters also make substantial contributions to carbon transport, mineralization, and sequestration (Battin et al., 2009; Seelen et al., 2019). An important process driving fluxes in the global carbon cycle is the microbial decomposition of dead organic material. The global annual carbon flux from terrestrial soils through microbial respiration is estimated to be about 68 Pg C year^−1^ (Raich & Schlesinger, 1992) and mineralization of the annual leaf litter fall accounts for ca. 50% of the CO_2_ output from soils (Coûteaux et al., 1995). Leaf litter input is also an important source of carbon in freshwater systems (Stoler & Relyea, 2020). Environmental conditions such as moisture and temperature must be considered as they influence terrestrial and aquatic decomposition (Djukic et al., 2018; Zhang et al., 2008). There are several definitions of decomposition: for example, Gessner et al. (2010) define decomposition as: “all biological processes contributing to organic matter mass loss and transformation, and not including physical losses caused by abrasion, fragmentation or leaching,” while Coûteaux et al. (1995) define it as: “(1) the concomitant mineralization and humification of lignin, cellulose and other compounds by a succession of microorganisms; and (2) the leaching downward in the soil of soluble compounds whose C and nitrogen (N) are progressively mineralized and immobilized.” Given that there exist different definitions, there seems to be a need for researchers to explicitly state which definition they use in their study.

Ecological studies often estimate decomposition (with and without leaching) of leaf litter using litterbags, i.e., bags made of non‐biodegradable mesh fiber that are filled with a known amount of plant litter from one or several species (e.g., Kampichler & Bruckner, 2009). When mesh sizes are small (0.25 mm), the results from such studies shed light on the determinants of microbial litter decomposition rates, such as latitude, climate (regional and seasonal), and litter quality (Althuizen et al., 2018; Zhang et al., 2008). Litter quality is strongly related to litter diversity effects such as complementarity (Gessner et al., 2010; Handa et al., 2014), which denotes mechanisms leading to higher decomposition rates of litter mixtures that cannot be explained by summing the decomposition rates of the single litter species involved. Since it is a challenge to separate the effects of environmental factors, especially climate, on decomposition from confounding litter quality effects (e.g., Keuskamp et al., 2013; Tiegs et al., 2019), there have been several attempts to use standardized substrates (see, e.g., Fritz et al., 2011; Tiegs et al., 2013). Consequently, Keuskamp et al. (2013) proposed the use of standardized, commercially available tea bags as a substrate to measure litter decomposition.

The Tea Bag Index (TBI) involves two standardized types of tea with different levels of decomposability, i.e., different fractions of labile and recalcitrant carbon compounds (Sarneel & Veen, 2017). As each tea portion is packaged within a fine mesh synthetic tea bag, mass loss by the teas over 90 days of incubation in the field is used to estimate the decomposition rate (*k*) and stabilization factor (S). The TBI presents a valuable methodological advancement for decomposition studies by removing variability due to differences in the local litter, facilitating comparison among biomes, ecosystems, and soil types while remaining a relatively cheap, easy‐to‐use method suitable for citizen‐science projects (Sandén et al., 2020). The TBI is being applied all over the globe, as exemplified by a large study summarizing early‐stage litter decomposition at 336 sites (Djukic et al., 2018), which found that, for example, mean annual precipitation had significant effects on decomposition across biomes. Several studies using the TBI method only report the percentage of mass loss over the incubation period as a measure of decomposition (e.g., Helsen et al., 2018; Houben et al., 2018; Marley et al., 2019). However, reporting mass loss as a proxy for decomposition does not account for the potential loss of litter mass due to non‐biotic initial leaching from the tea bags, which would relate to environmental variation in, for example, precipitation, snowmelt, or groundwater flux. Chemical analyses have shown that both green and rooibos tea have considerable water‐soluble fractions (0.493 and 0.215 g^−1^, respectively; Keuskamp et al., 2013), which are referred to as the hydrolyzable fraction when *k* and S are calculated. However, several authors (e.g., Cotrufo et al., 2010; Gessner et al., 1999; MacDonald et al., 2018) have noted the problem of an initial large amount of leaching (within the first 60 min) of the soluble fraction from litterbags or tea bags, and especially in very wet habitats (Marley et al., 2019). This mass loss might erroneously be attributed to microbial decomposition, although studies of colonization dynamics of leaf litter through fungi and bacteria show that these organisms need a few hours to colonize the incubated substrate (Krevš et al., 2017). For lakes, an adjusted equation has been suggested to account for the initial leaching (Seelen et al., 2019). Other studies have also shown differences in mass loss between pre‐leached and unleached tea bags (Blume‐Werry et al., 2021; Pouyat et al., 2017). Hence, when assessing microbial decomposition, ignoring leaching variability as a source of mass loss in environments with large variations in moisture will result in the overestimation of decomposition through microbial activity by bacteria and fungi in both aquatic and terrestrial habitats.

The aim of the present study was to quantify the initial mass loss through leaching, which happens within the first minutes and hours before the colonization of organisms, in habitats with different moisture and temperature for green and rooibos teas used in the TBI. We did this by contrasting the long‐term mass losses (90 days) under field conditions in both terrestrial and aquatic environments, to the short‐term losses (in the range of minutes and hours) due to leaching under field and laboratory conditions.

Specifically, we addressed the following questions:

1. How do water temperature and water volume affect the mass loss of green and rooibos tea during initial leaching periods under laboratory conditions?
2. What proportion of green and rooibos teas are lost through initial leaching in terrestrial habitats with varying soil water contents and in an aquatic habitat?
3. What proportion of the mass loss reported in TBI studies using 90‐day incubation times can be attributed to initial leaching and how does initial leaching‐derived mass loss affect the calculation of the decomposition rate (*k*) and stabilization factor (S) in different habitats?

By addressing these questions, we hope to provide new information that will help to improve the currently established TBI protocol and contribute to some understanding about leaching when estimating microbial litter decomposition. Finally, we want to address the current possibility of misunderstanding as it is not always explicitly stated what kind of decomposition is in focus in different studies.

## 2 MATERIALS AND METHODS

The tea bags used in each experiment were consistent with the recommendations on the TBI website (www.teatime4science.org) – green tea: Sencha exclusive collection EAN 8714100770542; rooibos tea: Rooibos and hibiscus infusion EAN 8722700188438. Prior to each experiment, we labeled pairs of green and rooibos nylon tea bags with unique codes and weighed each tea bag to the nearest 0.0001 g. Initial tea mass of air‐dried bags averaged 1.799 g (SD = 0.045) and 1.955 g (SD = 0.041) for green and rooibos tea, respectively, which is consistent with the masses given by Keuskamp et al. (2013); 1.773 g and 1.907 g, respectively.

### 2.1 Varying temperatures and volume in the laboratory

In a laboratory experiment, we tested how quickly the tea bags lose their hydrolyzable fraction depending on water temperature and volume (via water changes at regular intervals). To determine the effect of temperature and water changes on the extent of leaching from green and rooibos tea over time, we submerged tea bags (*n* = 6) from each tea in 0.33 L of regular tap water. Half of the tea bags were allowed to leach for a range of times (10, 20, 30, 40, 60, 80, or 180 min) and at a range of temperatures (8, 19, or 60°C). These temperatures were selected to mimic conditions observed during our field trials (8°C), standard room temperature (19°C), and hot tap water as an extreme value (60°C). The other half of the tea bags (*n* = 3 per trial) experienced the same conditions as above but were transferred to a new beaker containing 0.33 L of fresh tap water at the appropriate temperatures every 10 min. In this way, none of the tea bags from the second treatment remained in the same water for more than 10 min, thereby avoiding potential water saturation. In the treatment where tea bags were in the same water throughout the trial, the 8 and 19°C treatments held a constant temperature over the entire leaching period, whereas the 60°C treatment experienced a gradual drop in temperature over the course of the leaching period (a loss of 10°C over 180 min). No such decrease in temperature occurred when regular water changes were employed. After the treatments, all tea bags were air‐dried for at least 4 days before being reweighed.

### 2.2 Varying moisture levels in the field

To test the effect of moisture levels on the rate and extent of initial leaching, we chose several sites along the Alster River east of Karlstad, Sweden (59°24′09.72″N, 13°36′25.15″E). Two levels of soil moisture were sampled in terrestrial habitats adjacent to the river: moderate soil moisture (65%–75%) and high soil moisture (>90%) in addition to two fully aquatic habitats directly in the river (100% moisture). The sites contained typical boreal riparian vegetation with a combination of trees, bushes, grasses, herbs, and sedges (*Carex* spp.), and a silt‐clay soil type. The terrestrial samples were deployed on November 2nd, 2018, when air temperatures ranged from 5 to 9°C. We chose sample locations based on initial soil humidity levels measured using a moisture meter (type HH2 with a theta probe ML3: Delta‐T Devices Ltd, Cambridge, England). For the terrestrial habitats, three replicates of each variety of tea were used for each moisture level. Tea bags were buried ca. 8 cm deep in the soil and retrieved after 10, 20, 30, 60, 120, and 240 min. At the end of each leaching period, we dried the tea bags in an oven at 70°C for at least 48 h before weighing them.

The submerged aquatic samples were deployed on April 27th, 2018, within the Alster River, approximately 410 m downstream of the terrestrial sampling sites. At the selected sites, the river was 1.7–5.8 m wide and had a discharge between 0.04 and 1.83 m^3^/s (velocity 0.1–0.8 m/s) throughout the leaching period. The substrate within the river at these sites was a mixture of old bricks, cobbles, and detritus. The mean water temperature throughout the experiment was 6.0–6.5°C. For the aquatic habitat, we used six replicates of each type of tea. We immersed the tea bags and retrieved them after 5, 10, 15, 20, 25, 30, 45, 60, 120, and 240 min. To keep the tea bags submerged, we constructed a sampling device that sandwiched the tea bags between two sheets of chicken wire (13 mm mesh) firmly attached to one another with cable ties. During sampling, we anchored the sampling devices in the center of the river, no more than 1 m below the surface, as suggested by the NETLAKE protocol (https://nioo.knaw.nl/nl/netlake‐citizen‐science?qt‐netlake=4). As with the terrestrial samples above, we removed the appropriate tea bags from the water at the end of each leaching period and dried them in an oven at 70°C for a minimum of 48 h before reweighing them.

To test the effect of moisture levels on the rate and extent of decomposition over 90 days in terrestrial habitats, we chose several sites in mid‐Sweden (Appendix S1). Two levels of soil moisture were sampled in terrestrial habitats adjacent to rivers: moderate soil moisture (65%–80%) and high soil moisture (>90%). The sites had typical boreal riparian vegetation and a variation in soil types from fine to coarse sediment. Samples were buried in mid‐June and retrieved in mid‐September 2020. Soil humidity levels were measured using the above‐mentioned moisture meter. For the terrestrial riparian habitat, five replicates of each variety of tea were used for each moisture level. Tea bags were buried ca. 8 cm deep in the soil and retrieved after ~90 days after which they were dried and weighed. For an aquatic habitat, we used a site in the Mörrumsån River, southern Sweden (56°20′13.29″N, 14°42′02.00″E), with similar water velocities to those observed in our field experiment. Samples were deployed in early June 2019 and retrieved in September. Tea bags were placed into perforated plastic cups which were anchored to the river's bottom at depths ~1 m.

### 2.3 Statistical analysis

Mass loss data from the laboratory and field studies were first converted to percentages of the initial tea mass. The time series of mass loss data were then modeled using a variety of non‐linear, asymptotic curve functions, e.g., Michaelis–Menten, Gompertz, Hollings type III, and three‐parameter logistic, fit via least‐squares regression. Akaike's information criterion (AIC: lower AIC values indicate a model with better fit) values were used to identify the best fitting curve: a two‐parameter Michaelis–Menten curve (Equation (1)

(1) $$M_{t}=\frac{V_{\max}\times t}{\left(K_{m}+t\right)}$$

where *M*_*t*_ was the mass loss up to time *t*, *V*_max_ is the asymptotic maximum extent of leaching, and *K*_m_ (the Michaelis parameter) is the time required to reach 1/2 *V*_max_. Models were fit using non‐linear least‐squares regressions within the nlme package in R (Pinheiro et al., 2019). Separate parameter estimates (i.e., a separate curve) were fit for each treatment, using the starting parameter values of 30 for *V*_max_ and 10 for *K*_m_. Parameter estimates were then extracted along with 95% confidence intervals, calculated using a normal approximation to the distribution of the maximum likelihood estimators.

### 2.4 Application of results to long‐term terrestrial and aquatic TBI data

We employed the leaching parameter estimates (*V*_max_) in conjunction with a dataset where the TBI protocol was used in terrestrial environments with high soil moisture (65–80 and >90%) (dataset Lind & Watz, 2022). Average mass loss was calculated for 10 locations in the riparian zone of rivers in mid‐Sweden. The soil *V*_max_ estimates for both the 65%–80% and >90% moisture levels in terrestrial environments were used to adjust the observed mass loss in this dataset. We then calculated *k* and *S* estimates before and after taking leaching into account.

We also employed the leaching parameter estimates (*V*_max_) in conjunction with a dataset where the TBI protocol was used in an aquatic environment (dataset: Harbicht, 2019). Average mass loss over 90 days was calculated for the site in the Mörrumsån River. We used *V*_max_ estimates from the 100% moisture treatment to adjust the observed mass loss values to take leaching into account. Then, we calculated the average mass loss. As with above, *k* and *S* estimates were calculated before and after taking leaching into account.

## 3 RESULTS

### 3.1 Temperature influence on leaching in the laboratory

**FIGURE 1:**
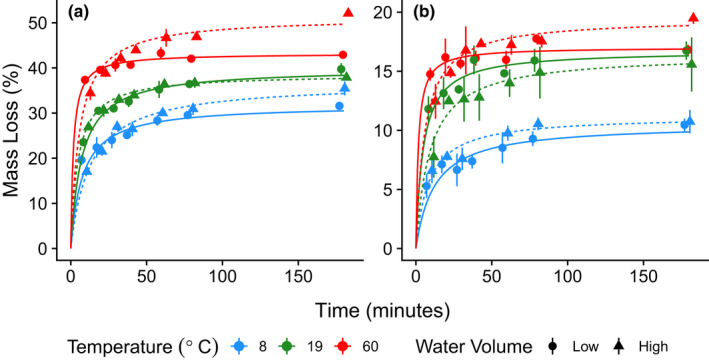
Michaelis–Menten curves fit separately, via least‐squares regression, to mass loss data due to leaching for green (a) and rooibos (b) tea over a range of water temperatures and conditions. Water conditions consisted of either constant water (0.3 L) over the entire study period or regular water changes at 10‐min intervals. Plotted points represent mean ± SE values with sample sizes of *n* = 3.

The observed mass loss from the rooibos tea ranged from 7.1 to 23.9% with a mean of 16.7%, while mass loss from the green tea ranged from 13.8 to 53.8% with a mean of 37.4% for all replicates in the different treatments (dataset, Lind et al., 2019). Both teas demonstrated a fast initial leaching rate, which slowed over time, eventually reaching an asymptotic maximum within the study period. This pattern was well suited for the Michaelis–Menten curve and the model residuals were minimal (residual SE = 1.18, d.f. = 25, Figure 1).

**FIGURE 2:**
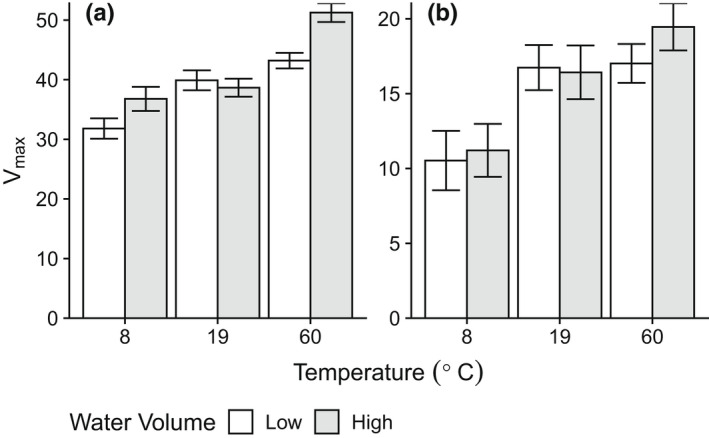
Parameter estimates ±95% confidence intervals for the maximum extent of leaching (*V*_max_) over a 180‐minute period from fitted Michaelis–Menten curves for green (a) and rooibos (b) tea. Teas were leached in tap water at a range of temperatures and water conditions: either constant (water change = no) or with regular water changes at 10‐min intervals.

With green tea, both water temperature and volume (water changes) influenced the estimated maximum extent of leaching (*V*_max_). Increasing the water temperature from 8 to 60°C resulted in an overall increase of 11.4 percentage points in the estimated maximum extent of leaching, from 31.81% to 43.20% under constant water conditions (Table 1, Figure 2a). Regularly changing the water at 10‐min intervals increased this overall range to 14.5 percentage points, from 36.78% to 51.25% over the same temperature range. Within water treatments, when a constant 0.33 L of water was used throughout the whole experiment, the largest increase in *V*_max_ (8.1 percentage points) occurred at lower temperatures, between 8 and 19°C. When the water volume was regularly changed every 10 min, the greatest increase in *V*_max_ (12.6 percentage points) occurred at higher temperatures, between 19 and 60°C. Within temperature treatments, changing the water at 10‐min intervals resulted in higher *V*_max_ estimates and non‐overlapping confidence intervals in two of three temperature levels: 8 and 60°C (Table 1, Figure 2a), but no noticeable effect at 19°C. Overall, the time required to reach 1/2 *V*_max_ (the *K*_m_ parameter) showed considerable overlap among temperature and water treatments and ranged from a minimum of 1.67 min when exposed to water at the hottest temperature (60°C) without water changes, to 12.89 min at the coldest temperatures (8°C) with water changes (Table 1).

**TABLE 1:** Parameter estimates from Michaelis–Menten kinetics curves fit to mass loss over time for green and rooibos teas under a range of conditions

Tea	Water conditions	Temperature (°C)	*V*_max_	95% CI	*K*_m_	95% CI
Green	Changing	8	36.78	34.76–38.8	12.89	10–15.78
		19	38.65	37.14–40.16	4.75	3.41–6.08
		60	51.25	49.68–52.82	5.7	4.58–6.82
	Constant	8	31.81	30.11–33.51	7.75	5.53–9.97
		19	39.9	38.23–41.57	7.24	5.55–8.92
		60	43.2	41.89–44.5	1.67	0.87–2.46
Rooibos	Changing	8	11.21	9.44–12.98	8.5	1.77–15.24
		19	16.42	14.63–18.22	9.29	4.37–14.2
		60	19.46	17.89–21.04	5.74	2.77–8.71
	Constant	8	10.53	8.55–12.51	12.2	2.6–21.81
		19	16.74	15.23–18.25	4.78	1.68–7.88
		60	17.02	15.72–18.32	1.57	−0.43–3.56

*Note*: The *V*_max_ parameter represents the maximum asymptotic mass loss due to leaching as a percentage of the initial mass. The *K*_m_ parameter represents the time required for the mass loss to equal half of *V*_max_, the Michaelis–Menten parameter, and CI is the confidence interval.

With rooibos tea, *V*_max_ estimates indicated no consistent effect of water changes on the maximum extent of leaching over the experimental range of temperatures: 95% confidence intervals overlapped for all three temperature treatments (8, 19, and 60°C). Within water treatments, however, the temperature did have a noticeable effect, with *V*_max_ estimates increasing with temperature. There was a greater effect of temperature on *V*_max_ estimates between 8 and 19°C under constant water than between 19 and 60°C (Table 1, Figure 2b), as the extent of leaching increased by 6.21 percentage points, from 10.53% to 16.74%. A similar increase was observed when the water was changed at regular 10‐min intervals. Hence, the 95% confidence intervals overlapped for both water treatments between the 19 and 60°C treatments, while the relative difference was much larger between 8 and 19°C. The speed at which the rooibos tea lost mass due to leaching (the *K*_m_ parameter) displayed considerable overlap (Table 1), ranging from 1.57 min at the hottest temperature (60°C) without water changes to 12.2 min under the coldest temperatures (8°C) without water changes.

### 3.2 Influence of moisture on the initial leaching process in the field

**FIGURE 3:**
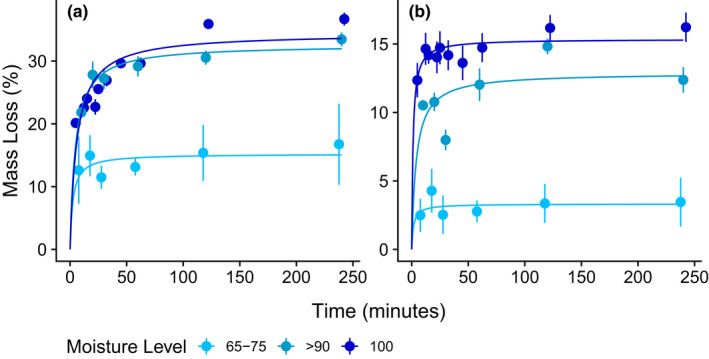
Michaelis–Menten curves fit via least‐squares regression, separately, to leaching data from green (a) and rooibos (b) tea placed in multiple environments in the field with varying moisture levels. Plotted points represent mean mass loss measurements ± standard errors.

**FIGURE 4:**
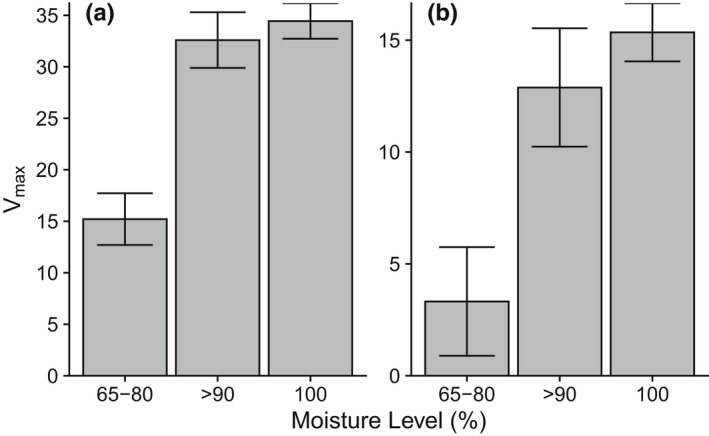
Parameter estimates ±95% confidence intervals for the maximum extent of leaching for green (a) and rooibos (b) tea in terrestrial and aquatic environments over a range of moisture levels (65–75, >90 and 100%).

The estimated maximum mass lost (*V*_max_) for green tea over a range of moisture levels in the instant field tests increased by 19.23 percentage points, from 15.21% to 34.44%, with the greatest differences occurring at lower moisture ranges, i.e., between the 65%–75% group and the 90% group (Table 2, Figure 3a,b). With rooibos tea, our experimental range of environmental moisture levels increased the estimated maximum mass loss by 12.03 percentage points, from 3.32% at 65–75% moisture to 15.35% at 100% moisture. With both tea litter types, increasing moisture levels from 65%–75% to 90% produced a significant increase in the *V*_max_ estimates, while 95% confidence intervals overlapped considerably between the 90 and 100% samples (Figure 4). Under these conditions, green tea required between 2.5 and 6 min to reach half the maximum estimated extent of leaching (*K*_m_), while rooibos tea required only between 1.2 and 4 min to reach the halfway point (Table 2). For each tea litter type, however, there was considerable overlap with the confidence intervals across environments for the *K*_m_ parameter.

**TABLE 2:** Parameter estimates for Michaelis–Menten curves fit to leaching data for green and rooibos tea incubated in the field (LOCATION) for 240 min in varying soil moisture levels.

Tea	Location	Moisture level	*V*_max_	95% CI	*K*_m_	95% CI
Green	Terrestrial	65–75	15.21	12.7–17.72	2.5	−2.05–7.05
		90	32.59	29.89–35.3	4.76	1.97–7.56
	Aquatic	100	34.44	32.72–36.17	6.03	4.5–7.55
Rooibos	Terrestrial	65–75	3.32	0.89–5.75	1.62	−16.81–20.05
		90	12.88	10.24–15.53	4.05	−2.46–10.57
	Aquatic	100	15.35	14.05–16.65	1.17	−0.24–2.59

*Note*: *V*_max_ = the asymptotic maximum extent of leaching, CI = confidence interval, *K*_m_ = (the Michaelis parameter) is the time required to reach 1/2 *V*_max_.

### 3.3 Leaching vs. microbial decomposition

**FIGURE 5:**
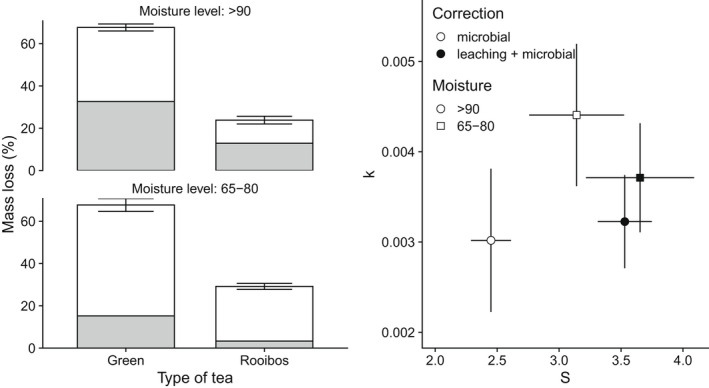
Bars in the left panel show average mass loss (%) of tea bags (green and rooibos) after 90 days of incubation in boreal riparian habitats. Shaded regions indicate the extent of mass loss (%) by leaching (*V*_max_) after 4 h from our field data at 65%–80% and >90% soil moisture, respectively. The right panel shows estimates of decomposition rate (*k*) and stabilization factor (*S*) based on the original data including leaching (black dot) and after correcting for mass loss due to initial leaching (microbial decomposition only) assuming moisture levels of 65%–80% (squares) and >90% (dot).

**FIGURE 6:**
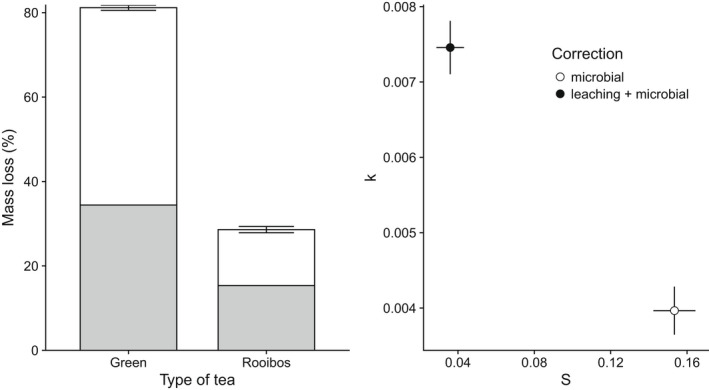
Average mass loss (%) ± standard deviation of tea bags (green and rooibos) after 90 days of incubation in the Mörrumsån River. Shaded region indicates the extent of mass loss (%) by leaching (*V*_max_) after 4 h from our field data (moisture level 100%). The right panel shows estimates of mean (±SE) decomposition rate (*k*) and stabilization factor (*S*) based on the decomposition including leaching (uncorrected: filled circle) and microbial decomposition (corrected: empty circle) estimates of mass loss.

From our estimates of the maximum extent of leaching in various terrestrial environments (65%–75% and >90% moisture), 15.2% to 32.6% of the mass loss attributed to decomposition of green tea is the result of leaching within the first 3–4 h of incubation, but with a distinct plateau after less than 1 h. We related these findings to the results of a 90 days decomposition study. We found a 32.6% and 12.9% mass loss due to leaching of the total estimated decomposition of green and rooibos tea, respectively, at the >90% soil moisture level. At 65%–80% soil moisture, leaching represented 15.2% and 0.3% of the total mass loss attributed to decomposition of green and rooibos tea, respectively (Figure 5). Comparing estimates of the decomposition rate (*k*) and the stabilization factor (*S*) calculated with and without leaching showed a considerable effect of adjusting for leaching. At 90% soil moisture levels, in boreal riparian habitats, the estimate of *k* decreased by 6.5% when mass loss estimates were adjusted to account for leaching (from 3.2 × 10^−3^ to 3.0 × 10^−3^). Conversely, estimates of S decreased by 30.9% when leaching was accounted for (from 3.53 to 2.45; Figure 5). When comparing measured leaching (within 4 h) and microbial decomposition from tea bags submerged in the stream for 3 months, we found that at least 47.1% and 39.9% of the mass of rooibos and green tea, respectively, were lost by leaching (Figure 6). When this correction factor was applied to the *k* and *S* estimates, the resulting values were 47% lower in the case of the *k* value and over three and a half times larger in the case of S (Figure 6).

## 4 DISCUSSION

Our results clearly show that the leaching of water‐soluble compounds accounts for a large extent of what mistakenly could be taken for decomposition through biological activity and that the extent of leaching depends on the moisture levels in the habitat of the study. In the laboratory part of our study, leaching increased with increasing temperature in both the standardized green and rooibos tea substrates used for estimating decomposition via the Tea Bag Index. Leaching further increased in two of the three temperatures for green tea when the water volume was increased through water changes. While additional water volume affected the extent of leaching during our lab study, it had little effect on the rate of leaching as a large proportion of the leaching happened very fast, and the shortest time to reach *K*_m_ (1/2 of maximum mass loss) for both tea litter types was found in treatments without any water changes. The field experiment indicated that initial leaching increases with soil moisture content in terrestrial habitats. In fact, in terrestrial soils with >90% moisture, leaching occurred to the same extent as in aquatic habitats. Thus, our study suggests that more specific and nuanced use of the term decomposition is needed to disentangle the initial leaching from the following microbial decomposition.

Previous studies on the leaching kinetics of leaf litter showed that there are large differences between fresh and dried leaves (Bärlocher, 1997; Gessner & Schwoerbel, 1989) for both deciduous and coniferous trees (Nykvist, 1963). Fresh leaves of alder and willow showed almost no mass loss during 10 days of immersion in water, while air‐dried leaves lost between 20 and 25% (Gessner & Schwoerbel, 1989). Furthermore, pre‐treating the litter through drying increased leaching in 18 of 27 species (Bärlocher, 1997), whereas fungal colonization and invertebrate consumption decreased in fresh compared to dried leaves. Consequently, Bärlocher (1997) and Gessner et al. (1999) argued that pre‐treatment may significantly affect mass loss through leaching and subsequent biotic decomposition of litter used in traditional litterbag studies. In addition to being dried, the standardized decomposition substrates of the Tea Bag Index have been pre‐treated by cutting the leaves (green tea) or bark (rooibos tea) into small pieces, significantly increasing the surface area of the teas. Ground leaves of deciduous and coniferous trees lost significantly higher proportions of their mass during the first day of immersion relative to intact leaves (Nykvist, 1963). Initial leaching of water‐soluble compounds may therefore be even higher in the tea bag decomposition substrates than for intact leaves of traditional litterbag studies. Also, Blume‐Werry et al. (2021) showed that the mass loss was higher from green tea (40%) compared to a mix of leaves from three naturally occurring species (*Alnus glutinosa*, *Lythrum salicaria*, and *Deschampsia cespitosa*), but lower than the mass loss from rooibos tea (20%). Thus, even though knowledge about the quantity of leaching losses exists, this is often not taken into consideration in studies where researchers use the percent biomass loss, rather than corrected values adjusting for moisture differences.

We assume that microbial decomposition is negligible during the first 3–4 h of immersion of tea bags in soil or water, which is consistent with colonization dynamics of leaf litter through fungi and bacteria (e.g., Krevš et al., 2017). Nevertheless, this timeframe is sufficient to capture the bulk of leaching losses as shown by our Michaelis–Menten model. Based on immersion (3 h) of 48 bags each of green and rooibos tea in the pelagic zone of a small pond in the Netherlands, Seelen et al. (2019) found an average mass loss of 28.0% for green and 11.3% for rooibos tea. Seelen et al. (2019) subsequently used these fractions as a correction factor for the calculation of *k* and S based on TBI mass loss in 40 lakes across Europe. Their leaching percentage is 0.82 (green tea) and 0.75 (rooibos tea) of our leaching losses assessed in the open water of a stream habitat, suggesting that water movement (volume) has an additional effect on the amount of mass loss by leaching. However, Seelen et al. (2019) only focused on lentic aquatic habitats while we present a gradient in moisture and its effects on the leaching process. Our study also suggests that the correction factor needs to be even higher than the one used by Seelen et al. (2019) to correct for leaching in fluvial environments compared to lakes.

When we relate our estimates of leaching in terrestrial habitats to mass loss of green and rooibos tea for riparian boreal sites over 90 days, leaching during the first 4 h may account for as much as 33% of total reported mass loss for green tea. For a stream dataset from the southern boreal region, about 50% of the mass loss over 90 days can be accounted for through leaching during the first 4 h of immersion. Not accounting for these large initial mass losses through abiotic leaching of water‐soluble compounds will lead to an overestimation of the microbial decomposition rate. Although decomposition rates calculated using tea bags were highly correlated with those estimated from litterbags (MacDonald et al., 2018), tea bags yielded systematically higher values of *k* compared to conventional litterbags in a study of decomposition in peatlands. Systematic overestimation of the decomposition rate may lead to erroneous results when using these values in subsequent large‐scale models, especially when only using the percent loss and not the calculated *k* and *S* values. However, concerns have also been raised regarding the assumption that *S* is equal for green tea and rooibos (Mori et al., 2022). Within multisite studies, ignoring differences in leaching losses will lead to higher mass loss measurements and an overestimation of microbial decomposition rates in wet environments compared with drier sites. For example, Djukic et al. (2018) found that mass loss significantly increased with mean annual precipitation across biomes. Warm and humid climates may have higher decomposition rates than dry and/or cold biomes, as observed/stated, but part of this relationship may be due to an overestimation of microbial mass loss through the TBI due to increased leaching at higher temperatures and with higher moisture. We should therefore practice healthy skepticism of any significant relationships between processes that may affect leaching (i.e., precipitation) and microbial decomposition rates calculated via the TBI. Hence, the applicability of TBI and other types of litter bags depends on the research question and future studies should clearly state if they focus on microbial decomposition or decomposition including leaching (e.g., ecosystem decomposition). Blume‐Werry et al. (2021) conclude from their study, finding fast and substantial mass loss due to leaching, that pre‐treating (leaching) tea bags before field incubation is not necessary and decomposition rates can be compared between systems, as long as soil moisture ranges between 5 and 25%. This range is characteristic of sandy soils, while loamy soils may show water contents between 20 and 40%. Additionally, higher water contents can be found in seasonally wet soils, floodplains, riparian zones, or after heavy rainfalls or snowmelt. Taken together this implies that it is important to be specific on the detailed purpose of the study as we suggest.

Even though the Tea Bag Index provides a standardized material for examining decomposition across climate regions, our study suggests that it is advantageous to differentiate the different parts of the decomposition process, e.g., leaching of water‐soluble compounds, fragmentation through detrivores, and chemical alteration through microorganisms (Aerts, 1997; Cotrufo et al., 2010). Regardless of which type of decomposition method is being used, it is important to take into consideration the major mass loss that occurs through the initial abiotic leaching, which in turn varies depending on moisture, habitat, and temperature.

## 5 CONCLUSIONS

Decomposition is fundamental to carbon, nutrient, and energy cycling among and within aquatic and terrestrial ecosystems. Understanding the rate of decomposition is therefore essential since it influences carbon storage, plant productivity, and species composition (Bradford et al., 2016; Parton et al., 2007). Our study shows that mass loss through abiotic leaching is currently not clearly accounted for and communicated in decomposition studies. Thus, to improve reliability and ensure comparable data, we encourage (1) the use of correction factors that is moisture/habitat dependent, and (2) a consistent use of *k* and *S*, and not mass loss in percentage.

## AUTHOR CONTRIBUTIONS

**Lovisa Lind:** Conceptualization (equal); data curation (lead); formal analysis (supporting); investigation (equal); methodology (equal); writing – original draft (lead); writing – review and editing (equal). **Andrew Harbicht:** Conceptualization (supporting); formal analysis (lead); investigation (equal); methodology (supporting); visualization (lead); writing – review and editing (equal). **Eva Bergman:** Conceptualization (equal); formal analysis (supporting); methodology (equal); supervision (lead); writing – review and editing (equal). **Johannes Edwartz:** Investigation (equal); writing – review and editing (supporting). **Rolf Lutz Eckstein:** Conceptualization (equal); formal analysis (supporting); methodology (supporting); supervision (supporting); writing – review and editing (equal).

## CONFLICT OF INTEREST

None to declare.

## Supporting information


Appendix S1

## Data Availability

All tea bag data can be accessed at Zenodo.org: [dataset] Harbicht (2019), Lind et al. (2019), and Lind and Watz (2022).
