# Supplementary material for: Effects of initial leaching for estimates of mass loss and microbial decomposition—Call for an increased nuance
Source: Ecol Evol. 2022 Jul 31;12(8):e9118. doi: 10.1002/ece3.9118 (PMC9339754; doi:10.1002/ece3.9118)
Supplement: Supplementary file 1 — Appendix S1 [file ECE3-12-e9118-s001.docx]

APPENDIX 1.

TABLE A1. Riparian sites used for the 90 days decomposition part of the study, three samples were deployed at along Röjdan.

| Site | Longitude | Latitude | Elevation (m a.s.l.) |
| --- | --- | --- | --- |
| Karåsforsen | 14°59'70"E | 59°36'98"N | 134 |
| Gullspång | 14°11'09"E | 58°98'74"N | 67 |
| Lima | 13°42'48"E | 60°78'71"N | 335 |
| Eldforsen | 14°23'79"E | 60°44'26"N | 241 |
| Blyberg | 14°16'22"E | 61°14'74"N | 269 |
| Dals Långed | 12°30'18"E | 58°92'30"N | 66 |
| Malfors | 15°50'45"E | 58°52'14"N | 96 |
| Röjdan | 12°96'81"E | 60°13'46"N | 89 |
